# Editorial: Problematic Internet Technology Use: Assessment, Risk Factors, Comorbidity, Adverse Consequences and Intervention

**DOI:** 10.3389/fpsyt.2021.786019

**Published:** 2021-10-22

**Authors:** Jon D. Elhai, Dmitri Rozgonjuk, Julia Brailovskaia

**Affiliations:** ^1^Department of Psychology, University of Toledo, Toledo, OH, United States; ^2^Department of Psychiatry, University of Toledo, Toledo, OH, United States; ^3^Department of Molecular Psychology, Institute of Psychology and Education, Ulm University, Ulm, Germany; ^4^Institute of Mathematics and Statistics, University of Tartu, Tartu, Estonia; ^5^Mental Health Research and Treatment Center, Department of Clinical Psychology and Psychotherapy, Ruhr-Universität Bochum, Bochum, Germany

**Keywords:** internet addiction, smartphone addiction, problematic internet behavior, mental health, smartphone

There is no doubt that many personal and societal advantages are associated with using Internet technology such as social networking sites (SNS), gaming, and smartphones. For instance, smartphones have enhanced productivity in workplace (1) and educational (2) settings, and can facilitate health and mental health treatment with apps designed to complement traditional interventions (3). Furthermore, using SNS can boost social capital (4, 5), which can in turn promote mental health (6, 7). Such advantages of Internet technology use are relevant when such use is of mild to moderate frequency, conducted in healthy and adaptive ways. However, Internet technology is a double-edged sword, and can alternatively be used in unhealthy, maladaptive ways (8, 9).

In the current Research Topic, we address when Internet technology is used in ways that are problematic or excessive, causing dysfunction in daily life. Problematic use of Internet technology is influenced by risk factors such as mental health symptoms (10–12) which drive such problematic use in an effort to alleviate negative affect (13, 14). Additional risk factors for problematic Internet use involve predispositional characteristics such as personality, genetics and other biological factors, deep seated cognitions (14–16), as well as cognitive and affective responses and dysfunctional coping processes (17, 18). In fact, theoretical models have been developed and supported that discuss how this variety of risk factors may contribute to problematic Internet use (19). Furthermore, consequences of problematic Internet use include physical pain in the hands and neck (20, 21), pedestrian and driving collisions (22), distraction and poor performance in school and work (23–25), and can involve cyberbullying (26), problematic pornography use (27), and internet radicalization (28).

In the present Research Topic, authors present research in several domains related to problematic Internet use. Several papers report the development and/or validation of scales used to measure aspects of problematic Internet use—including problematic use symptoms [(29), Paschke et al.] and distractions from the smartphone (Throuvala et al.). These papers also report how these scales are related to external constructs such as mental health symptoms. For instance, Burkauskas et al. discovered that the nine-item Problematic Internet Use Questionnaire was valid in a sample of Lithuanian residents, and correlated positively with mental health symptom severity. Paschke et al. found that their newly developed Social Media Use Disorder Scale for Adolescents correlated positively with severity of depression and stress in German adolescents. And Throuvala et al. discovered that their newly developed Smartphone Distraction Scale correlated positively with emotional dysregulation and problematic SNS use in a sample of British university students. Such studies are important in providing researchers and clinicians with valid assessment instruments for measuring problematic Internet use and its consequences.

Other authors in this Research Topic examined stress and anxiety as potential risk factors for the problematic use of Internet technology (Yang et al.; Li et al.; Zhao and Zhao). These papers also importantly examine potential mediators or moderators (mechanisms) that can explain how stress or anxiety are related to problematic Internet use, including the fear of missing out (FOMO) on rewarding experiences (Yang et al.), self-efficacy (Li et al.), and active SNS use or SNS flow (Zhao and Zhao). Examining such mechanisms is important because psychopathology alone may not adequately explain the development or maintenance of problematic Internet use (14, 15). For example, Yang et al. revealed that FOMO mediated relations between stress and problematic smartphone use severity in a sample of Chinese university students. Li et al. found support for self-efficacy in partially mediating associations between anxiety and problematic smartphone use symptoms in a sample of Chinese college students. And Zhao and Zhao discovered that active SNS use and SNS flow mediated relations between stress about COVID-19 and problematic SNS use in Chinese college students. We believe that future research should continue to prioritize testing of moderators and mediators that explain associations between both stress and anxiety with problematic Internet use.

Other papers examine additional risk factors for problematic Internet use. Guo et al. sampled Chinese residents using a population-based survey, and examined how using different features of the smartphone may relate not only to problematic use but also to its different facets. Schivinski et al., Zhang et al., and Heng et al. examined social-related variables in association with problematic Internet use. Specifically, Schivinski et al. used an English-speaking sample of SNS users, finding that particular social motives (especially intrapersonal) were related to problematic SNS use severity. Heng et al. used a Chinese sample of undergraduates, discovering that social capital mediated relations between within-game social interactions and problematic gaming. And Zhang et al. sampled participants from China and Germany, finding that autistic traits were related to problematic Internet use. Finally, Luo et al. sampled Chinese college students, finding that adaptability regarding emotions, homesickness, and learning were related to perceived distress from losing smartphone access (or nomophobia). Studying such risk factors as social-related variables, autistic traits, and adaptability are important in furthering our understanding of why some people excessively engage in Internet use.

Finally, we mention the important commentary by Montag and Hegelich. The authors present a compelling argument that an important determinant of problematic SNS use is the way in which SNSs were developed and operate financially. That is, it is the intention of SNSs to prolong users' SNS use in order to use their data and profit from it. The authors also discuss significant societal adverse effects from the SNS business model, including problematic use, privacy infringement, and impingement on democracy through the spread of fake news.

To conclude, the present Research Topic provides readers with recent cross-national findings considering the assessment of different forms of problematic Internet technology use, and the complex mechanisms underlying the association between problematic Internet technology use and mental health that involve different inter- and intrapersonal as well as environmental and societal factors. The works published in this Research Topic contribute to the understanding of Internet technology use associations with daily-life adversities and may be useful for professionals working in this line of research.

## Author Contributions

All authors wrote and edited this paper.

## Conflict of Interest

The authors declare that the research was conducted in the absence of any commercial or financial relationships that could be construed as a potential conflict of interest.

## Publisher's Note

All claims expressed in this article are solely those of the authors and do not necessarily represent those of their affiliated organizations, or those of the publisher, the editors and the reviewers. Any product that may be evaluated in this article, or claim that may be made by its manufacturer, is not guaranteed or endorsed by the publisher.
